# The effects of organic and inorganic phosphorus amendments on the biochemical attributes and active microbial population of agriculture podzols following silage corn cultivation in boreal climate

**DOI:** 10.1038/s41598-019-53906-8

**Published:** 2019-11-21

**Authors:** Waqas Ali, Muhammad Nadeem, Waqar Ashiq, Muhammad Zaeem, Syed Shah Mohioudin Gilani, Sanaz Rajabi-Khamseh, Thu Huong Pham, Vanessa Kavanagh, Raymond Thomas, Mumtaz Cheema

**Affiliations:** 1 School of Science and the Environment, Grenfell Campus Memorial University of Newfoundland, Corner Brook, Newfoundland and Labrador A2H 5G4 Canada; 2 Shahrekord University, Rahbr Blvd, Shahrekord Chaharmahal and Bakhtiari, Shahrekord, Iran; 3 Department of Fisheries and Land Resources, Government of Newfoundland and Labrador, Pasadena, A0L 1K0 Canada

**Keywords:** Plant physiology, Plant stress responses

## Abstract

Phosphorus (P) is the second most important macronutrient that limits the plant growth, development and productivity. Inorganic P fertilization in podzol soils predominantly bound with aluminum and iron, thereby reducing its availability to crop plants. Dairy manure (DM) amendment to agricultural soils can improve physiochemical properties, nutrient cycling through enhanced enzyme and soil microbial activities leading to improved P bioavailability to crops. We hypothesized that DM amendment in podzol soil will improve biochemical attributes and microbial community and abundance in silage corn cropping system under boreal climate. We evaluated the effects of organic and inorganic P amendments on soil biochemical attributes and abundance in podzol soil under boreal climate. Additionally, biochemical attributes and microbial population and abundance under short-term silage corn monocropping system was also investigated. Experimental treatments were [P_0_ (control); P_1_: DM with high P_2_O_5_; P_2_: DM with low P_2_O_5_; P_3_: inorganic P and five silage-corn genotypes (Fusion RR, Yukon R, A4177G3RIB, DKC 23-17RIB and DKC 26-28RIB) were laid out in a randomized complete block design in factorial settings with three replications. Results showed that P_1_ treatment increased acid phosphatase (AP-ase) activity (29% and 44%), and soil available P (SAP) (60% and 39%) compared to control treatment, during 2016 and 2017, respectively. Additionally, P_1_ treatments significantly increased total bacterial phospholipids fatty acids (ΣB-PLFA), total phospholipids fatty acids (ΣPLFA), fungi, and eukaryotes compared to control and inorganic P. Yukon R and DKC 26-28RIB genotypes exhibited higher total bacterial PLFA, fungi, and total PLFA in their rhizospheres compared to the other genotypes. Redundancy analyses showed promising association between P_1_ and P_2_ amendment, biochemical attributes and active microbial population and Yukon R and DKC 26-28RIB genotypes. Pearson correlation also demonstrated significant and positive correlation between AP-ase, SAP and gram negative bacteria (G^−^), fungi, ΣB-PLFA, and total PLFA. Study results demonstrated that P1 treatment enhanced biochemical attributes, active microbial community composition and abundance and forage production of silage corn. Results further demonstrated higher active microbial population and abundance in rhizosphere of Yukon R and DKC 26-28RIB genotypes. Therefore, we argue that dairy manure amendment with high P_2_O_5_ in podzol soils could be a sustainable nutrient source to enhance soil quality, health and forage production of silage corn. Yukon R and DKC 26-28RIB genotypes showed superior agronomic performance, therefore, could be good fit under boreal climatic conditions.

## Introduction

Fertilizers are crucial inputs affecting the soil fertility, health and quality to optimize agricultural production. However, the minimal inputs of organic nutrient sources and dependency on inorganic fertilizers generally reduce soil organic carbon (SOC) and total microbial biomass (TMB) resulting in reduced soil health and overall crop productivity^1^. Therefore, to uplift the soil health and physiochemical properties, management practices such as manure application, crop residue incorporation^2–4^. Sustainable cropping systems involves the integrated use of organic (manure or crop residues) and inorganic fertilizer inputs to improve the ecosystem functioning and microbial activities in nutrients provisioning to growing plants^5^. For example, dairy manure (DM) and inorganic fertilizer used as organic and inorganic soil amendments can restore SOC and soil health^6,7^. Organic amendments substantially improve the soil C retention resulting in higher crop harvests^8^, retain C in the surface soil, and increase crop yields^9^. Furthermore, soil organic amendments could reduce the dependency on inorganic fertilizers^10^. Thus, amending soil with appropriate organic mineral source could be a better strategy to improve soil C and soil health through active microbial community structure under low fertility as well as in shallow soils^4,11^. DM and other organic mineral sources provide the essential plant nutrients through improved soil aggregations and aeration, adding organic matter (OM) and maintain soil pH particularly in acidic soils^12–14^.

Phosphorus (P) is the second important macronutrient limiting the plant growth and is least mobile nutrient plant rhizosphere^15^. Despite P being quite abundant in many soils, it is largely unavailable for plant uptake because P forms insoluble complexes with cations under acid and alkaline conditions. As a result, a large amount of inorganic P fertilizers has been applied to sustain agricultural production systems. Currently, global P fertilization is approximately 50 million tons with a projected annual increase of 20 million tons by 2030. It is also important to note the import of inorganic P fertilizers in areas such as Newfoundland where all inorganic P comes from mainland with significant dollar amount. Excessive P fertilization contributes to greenhouse gases and has a direct negative impact on surface waters that influence the functioning of ecosystems. This creates a challenge for the goal of increasing crop production while using inputs in such a way as to avoid environmental problems. Excessive or little P application led to severe and negative impacts on environment by causing by land degradation under P deficient conditions and eutrophication under excessive application^16^. The global crop productivity has strong relationship with soil fertility and most of the world soils are P deficient hence leading to lower crop productivity, however P deficiency is more often found in old weathered soils. Podzolic soils typically have a coarse-sandy texture and high acidity, with pH in the topsoil layer around 4 to 4.5^17^. These conditions signal a poor nutrient supply for agricultural crops. However, climatic changes might cause a northward shift of warmer weathers to provide enough crop heat units to exploit the agricultural resources, such as marginal lands in the boreal ecosystem characterized with podzol soils^18,19^. Such expansions of agricultural farmlands in the boreal ecosystem is one of the prime objectives to enhance the food production and food security; however, the marginal shallow agricultural lands with acidic soils are considered main limiting factor in production^20,21^. Hence, there is a need to investigate the effects of agricultural practices on soil health, quality and crop production for sustainable agriculture in the podzolic soils of the boreal ecosystem. Owing to P role in cell division as a component of nucleoproteins, it is involved in the cell reproduction, vegetative as well as reproductive development^22^. DM application increased soil acid phosphatase (AP-ase) and soil available phosphorus (SAP)^23,24^, and AP-ase is directly related to soil microbial activities^25^, and play major role in organic matter (OM) degradation, mineralization processes and plant nutrients availability^26^. TMB influences the physiochemical and biochemical soil properties; for instance, nutrient cycling and micro aggregation, thereby altering the soil biochemical environment and enhancing the crop production^1,27,28^. Hence, the presence of microbes and their richness is considered as a key tenet of soil quality in agricultural production systems. Different fertilization regimes affect soil microbial community (SMC) composition and structure, as bacteria is predominantly adapted to high available C and rich nutrient conditions whereas; fungi seem to be more adapted to recalcitrant C sources^29^. Different fertilization regimes affect SMC composition and structure, as bacteria is predominantly adapted to high available C and rich nutrient conditions, whereas, fungi seem to be more adapted in recalcitrant C sources^30,31^. Furthermore, chemical fertilizers applications may have potential effects on TMB and activities^32^. Very little is currently known concerning how DM and inorganic fertilizer amendment as P source affect soil biochemistry (enzymes, SAP, pH) and active microbial community composition in podzols used for agriculture production in boreal climates^33^.

Crop rotations could result in enhanced soil health in long-term agricultural production systems through regulating microbial community structures^17^. Such effects originated by selective root exudations by growing crop to build-up active microbes in rhizosphere^34–36^. There is substantial body of knowledge on effects of long-term mono-cropping, intercropping, crop rotation and cropping sequence on microbial community composition and abundance. However, there is a scarcity of information on the effects of short-term cultivations of silage corn on soil biochemical properties and the active microbial community composition and diversity in podzols under cool climates in boreal ecosystem^37^. Podzolic soils are coarse and sandy in texture with low pH^38^. Generally, the podzols are found in boreal ecosystem and temperate regions representing about 4% of total land surface area^39^. Podzolic soils are characterized humified OM in illuviated B horizons along with aluminum and iron, often seen as light colored Ae horizon^38^. Such conditions signal a poor nutrient supply and soil health for agricultural production systems. However, changing demands for agricultural land use due to climate-change and its northward shift necessitate the conversion of podzolic soils for agriculture production to meet the food security challenges in the 21^st^ century^21^. Considering podzols are not traditionally used for agriculture, there is limited knowledge related to how different management practices and genotypes affect the active soil microbial population and biochemical properties.

We hypothesized that organic and inorganic P amendments would change the soil biochemical attributes and health by modulating the active microbial composition and abundance and nutrient availability of silage-corn cultivated in boreal ecosystem. To test the hypothesis, a field experiment was conducted: (i) to investigate the effects of DM and inorganic P (IP) amendments on biochemical attributes and active microbial community composition and abundance (ii) to determine the response of silage corn genotypes cultivated as short term monocrop on the soil biochemical properties and the active microbial community composition and abundance amended with different P amendments.

## Results

### Biochemical attributes

Analysis of variance (ANOVA) showed that genotypes (G) and phosphorus amendments (P) interaction significantly (*p < 0.001*) influenced soil rhizosphere pH during 2016 and 2017 (Supplementary Information, Table S1). In 2016, higher pH was observed in the rhizosphere of Fusion RR genotype amended with inorganic P fertilizer, compared to lowest soil pH observed in the rhizosphere of DKC26-28RIB genotype. However, A4177G3 RIB and DKC23-17RIB genotype exhibited higher and lower soil rhizosphere pH following IP fertilizer in 2017 (Table 1). Phosphatase enzymes hydrolyze various organic and inorganic phosphate esters and played important role in the P-cycle. G × P interaction had significant (*p* < *0.001*) effects on AP-ase activity during 2016 (Table S1). Higher AP-ase was observed when Yukon R was amended with high P_2_O_5_ manure, whereas the lowest AP-ase was found in DKC 23-17RIB in control treatment (Table 1). In 2017, G × P interaction had no significant effects on AP-ase (Table S1). However, G × P interaction had significant (*p* < *0.01*) effects on SAP during 2016 (Table S1). Higher SAP level (129.36 mg/kg) was observed when Yukon R was amended with high P_2_O_5_ manure, and lowest (49.67 mg/kg) was found when A4177G3RIB genotype was cultivated in control treatment (Table 2). In 2017, G × P interaction had no significant effects on SAP whereas, genotypes and P sources individually had significant effects on SAP (Table S1). Among genotypes, higher SAP was noted in the rhizosphere of Yukon R while the lowest was found in DKC23-17RIB. Among P amendments, both high and low P_2_O_5_ manure applications resulted in higher SAP compared to the control treatment (*Supplementary information*, Table S2).

**Table 1:** Interactive effects of organic and inorganic phosphorus amendments and silage corn genotypes on soil pH. Each value represents mean of three replications.

		Fusion RR	Yukon R	A4177G3 RIB	DKC 23-17RIB	DKC26-28RIB	Means
2016	P_0_	6.09 ^ab^	6.09 ^ab^	5.92 ^efg^	5.98 ^cdef^	5.98 ^cdef^	6.01 ^A^
	P_1_	6.01 ^bcde^	6.06 ^abc^	5.96 ^defg^	5.95 ^defg^	5.96 ^defg^	5.99 ^AB^
	P_2_	6.03 ^abcd^	6.01 ^bcd^	5.95 ^defg^	5.89 ^fgh^	5.98 ^cdef^	5.97 ^AB^
	P_3_	6.12 ^a^	6.02 ^bcd^	5.92 ^efg^	5.88 ^gh^	5.83 ^h^	5.95 ^B^
	Means	6.06 ^A^	6.04 ^A^	5.94 ^B^	5.92 ^B^	5.94 ^B^	
2017	P_0_	6.18 ^abcde^	6.05 ^bcdefg^	5.91 ^efgh^	5.58 ^i^	5.73 ^hi^	5.89 ^C^
	P_1_	5.88 ^fgh^	6.29 ^ab^	6.28 ^abc^	6.20 ^abcd^	6.11 ^abcdef^	6.15 ^A^
	P_2_	6.10 ^abcdef^	5.97 ^defgh^	6.06 ^bcdef^	5.82 ^ghi^	5.93 ^defgh^	5.97 ^BC^
	P_3_	6.08 ^bcdefg^	6.13 ^abcdef^	6.38 ^a^	5.57 ^i^	6.00 ^cdefgh^	6.03 ^AB^
	Means	6.06 ^AB^	6.11 ^A^	6.15 ^A^	5.79 ^C^	5.94 ^B^	

Different superscripts indicate significant differences among treatments means at *p* < 0.05. P_0_: control, P_1_: manure with high P_2_O_5_ conc., P_2_: manure with low P_2_O_5_ conc., P_3_: inorganic P_2_O_5_. Fusion RR, Yukon R, A4177G3RIB, DKC 23-17RIB, DKC26-28RIB are silage corn genotypes used in this study.

**Table 2:** Variation in soil AP-ase and SAP with organic and inorganic phosphorus amendments and cultivated silage corn genotypes. Each value represents mean of three replications.

	Fusion RR	Yukon R	A4177G3 RIB	DKC 23-17RIB	DKC26-28RIB	
	Acid phosphatase activities					Means
P_0_	14.55 ^hij^	14.99 ^fgh^	13.95 ^j^	11.03 ^k^	14.25 ^ij^	13.75 ^C^
P_1_	17.15 ^cd^	19.07 ^a^	16.70 ^d^	17.85 ^bc^	17.91 ^b^	17.73 ^A^
P_2_	15.54 ^ef^	16.94 ^d^	15.30 ^efg^	15.68 ^ef^	15.93 ^e^	15.88 ^B^
P_3_	14.48 ^hij^	14.83 ^ghi^	14.70 ^ghi^	18.21 ^b^	15.84 ^e^	15.61 ^B^
Means	15.43 ^CD^	16.46 ^A^	15.16 ^D^	15.69 ^BC^	15.98 ^B^	
**Soil available phosphorus**						
P_0_	63.9 ^hi^	74.59 ^efg^	49.67 ^j^	56.59 ^ij^	57.95 ^ij^	60.54 ^D^
P_1_	93.65 ^cd^	129.36 ^a^	77.61 ^efg^	83.26 ^de^	100.78 ^bc^	96.93 ^A^
P_2_	75.82 ^efg^	107.79 ^b^	69.66 ^fgh^	78.31 ^efg^	96.45 ^c^	85.60 ^B^
P_3_	69.01 ^gh^	93.67 ^cd^	52.25 ^j^	68.97 ^gh^	80.03 ^ef^	72.78 ^C^
Means	75.59 ^C^	101.35 ^A^	62.3 ^D^	71.78 ^C^	83.8 ^B^	

Different superscripts indicate significant differences among treatments means at *p* < 0.05. P_0_: control, P_1_: manure with high P_2_O_5_ conc., P_2_: manure with low P_2_O_5_ conc., P_3_:Inorganic P. Fusion RR, Yukon R, A4177G3RIB, DKC 23-17RIB, DKC26-28RIB are silage corn genotypes used in this study.

### Total soil carbon and nitrogen

Genotypes and G x P interaction had nonsignificant effects on total soil C during 2016 and 2017 growing seasons, whereas organic and inorganic P amendments exhibited significant changes in total soil C (Table S1). In 2016, P_1_ and P_2_ treatments showed 21% and 16% higher total soil C than control (Table 3), whereas, 22% and 12% total soil carbon increase was observed in P_1_ and P_2_ treatments compared to control during 2017 (Table 3). Results further demonstrated that genotypes and P amendment exhibited significant effects on total soil N (Table S1). In 2016 and 2017, total soil N (0.31% and 0.33%) was higher in the rhizosphere of Yukon R, whereas lowest total soil N was observed in DKC 23-17 RIB (Table 4). In 2016, P_1_, P_2_ and P_3_ amendment exhibited 30%, 24% and 14% higher total soil N compared to control (Table 1), whereas during 2017, P_1_, P_2_ and P_3_ amendments showed 35%, 19% and 9% higher total soil nitrogen than control (Table 3).

**Table 3:** Effects of organic and inorganic phosphorus amendments on total soil nitrogen and carbon during 2016 and 2017.

	Phosphorus sources	Total nitrogen (%)	Total carbon (%)
	P_0_	0.24 ^c^	4.75 ^b^
2016	P_1_	0.32 ^a^	5.75 ^a^
	P_2_	0.30 ^ab^	5.49 ^a^
	P_3_	0.28 ^b^	5.09 ^b^
	P_0_	0.26 ^c^	4.83 ^c^
2017	P_1_	0.35 ^a^	5.88 ^a^
	P_2_	0.31 ^b^	5.42 ^ab^
	P_3_	0.29 ^bc^	5.11 ^bc^

Different superscripts indicate significant differences among treatments means at *p* < 0.05. P_0_: control, P_1_: manure with high P_2_O_5_ conc., P_2_: manure with low P_2_O_5_ conc., P_3_: inorganic P.

**Table 4:** Response of silage corn genotypes to total soil nitrogen and carbon in plants rhizosphere during 2016 and 2017 growing season.

	Genotypes	Total nitrogen (%)	Total carbon (%)
	Fusion RR	0.27 ^bc^	5.22 ^NS^
	Yukon R	0.31 ^a^	5.46
2016	A4177G3 RIB	0.28 ^abc^	5.21
	DKC 23-17 RIB	0.26 ^c^	5.06
	DKC 26-28 RIB	0.30 ^ab^	5.41
	Fusion RR	0.30 ^ab^	5.14 ^NS^
	Yukon R	0.33 ^a^	5.65
2017	A4177G3 RIB	0.29 ^b^	5.20
	DKC 23-17 RIB	0.27 ^b^	5.46
	DKC 26-28 RIB	0.31 ^ab^	5.13

Different superscripts indicate significant differences among treatments means at *p* < 0.05

### Soil PLFA profiles

**Figure 1:**
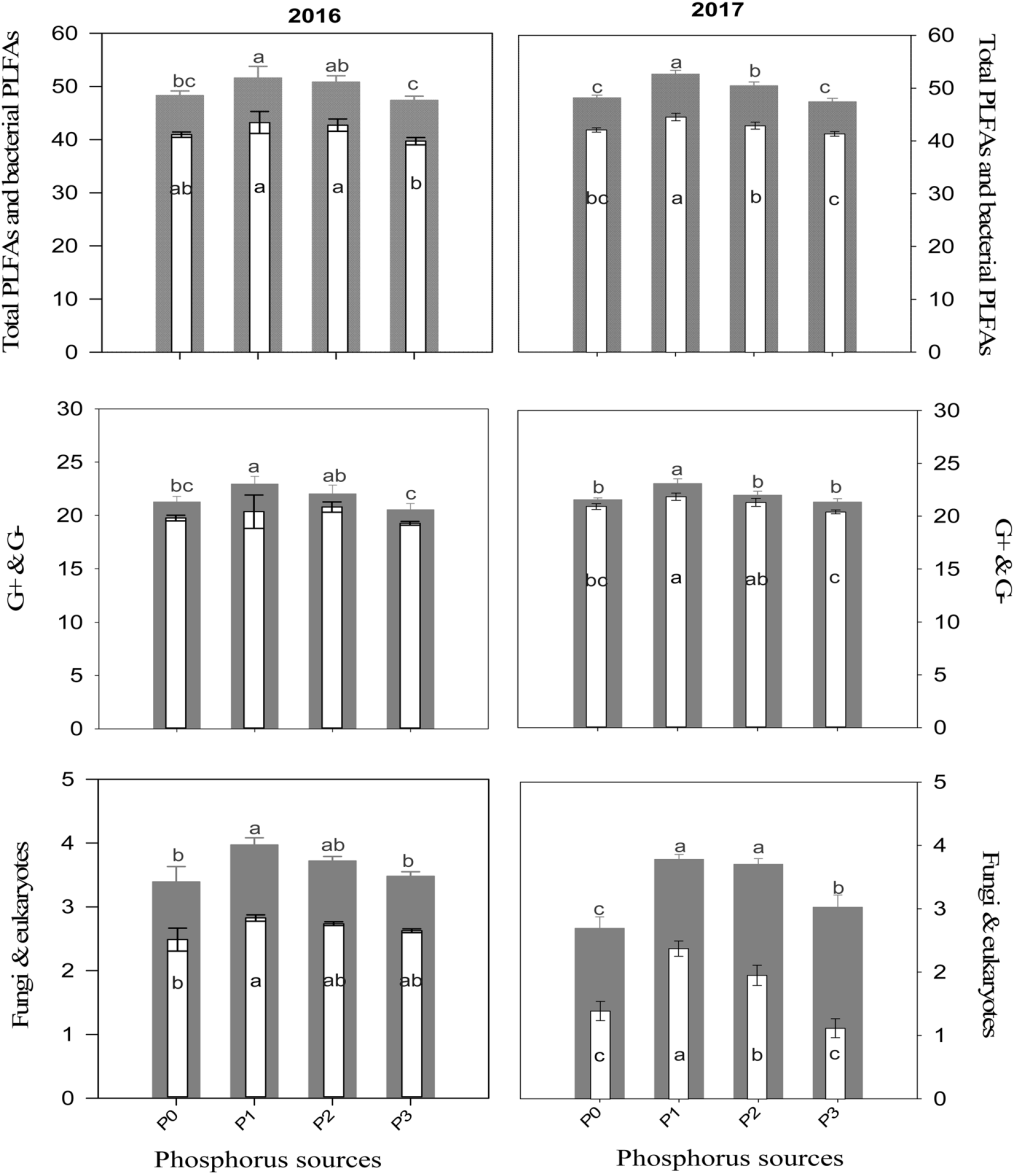
Variations of the total PLFA (gray wide bars), total bacterial PLFA (white narrow bars), gram negative bacteria (gray wide bars), gram positive bacteria (white narrow bars), fungi (gray wide bars) and eukaryotes (white narrow bars) under organic and inorganic phosphorus fertilizer amendments during 2016 (left) and 2017 (right). Error bars in each graph represents ± SE (n = 3). Different letters above bars indicate significant differences between organic and inorganic phosphorus sources at *P* < 0.05.

**Figure 2:**
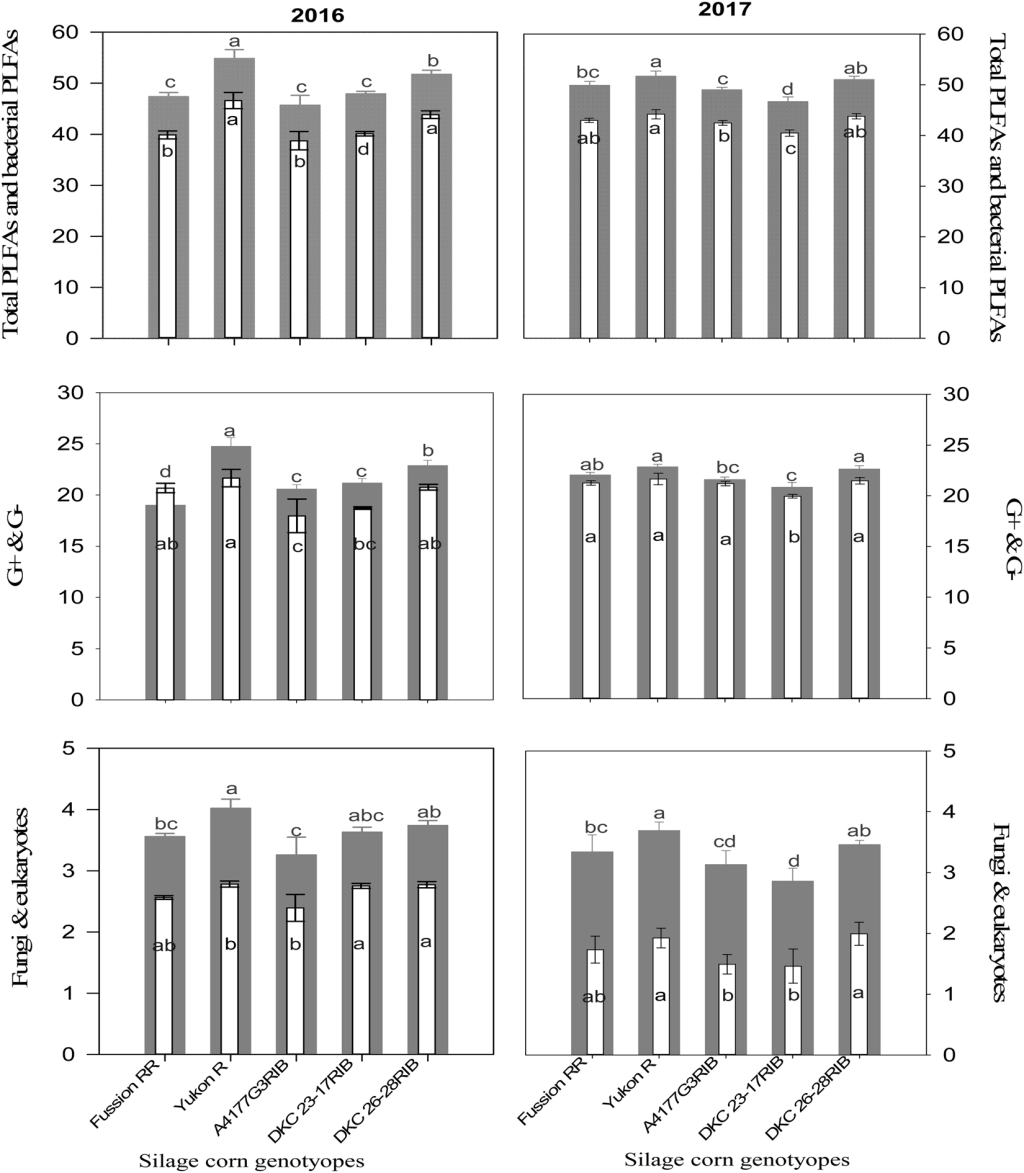
Variations of the total PLFA (gray wide bars), total bacterial PLFA (white narrow bars), gram negative bacteria (gray wide bars), gram positive bacteria (white narrow bars), fungi (gray wide bars) and eukaryotes (white narrow bars) in silage corn genotypes during 2016 (left) and 2017 (right). Error bars in each graph represents ± SE (n = 3). Different letters above bars indicate significant differences among silage-corn genotypes at *P* < 0.05.

ANOVA showed that G × P interaction had no significant effects on active soil microbial community during both years. However, P amendments had significant effects on total PLFA (ΣPLFA), total bacterial PLFA (ΣB-PLFA), gram negative (G^−^) bacteria, and fungi during 2016 and significantly affected ΣPLFA, ΣB-PLFA, fungi, G^−^, gram positive (G^+^) and eukaryotes during 2017 (Table S3). Silage corn genotypes also significantly affected the ΣPLFA, ΣB-PLFA, gram positive (G^+^), gram negative (G^−^) bacteria, fungi, eukaryotes, and G^+^:G^−^ ratio during 2016 and ΣPLFA, ΣB-PLFA, gram positive (G^+^), gram negative (G^−^) bacteria, fungi, and F:B ratio during 2017 (Table S3). P1 treatment (DM with high P_2_O_5_) enhanced ΣPLFA, ΣB-PLFA, G^+^, fungal PLFA, and eukaryotes compared to control and IP treatments during 2016, and increased G^−^ bacteria in addition to other microbial attributes mentioned above during 2017 (Fig. 1). Moreover, G × P interaction had significantly *(p* < *0.01)* affected fungi and F:B ratio during 2017 (Table S3). Higher fungal PLFA was observed in the rhizosphere of Yukon R amended with high P_2_O_5_ manure (Table S4). Regarding genotypes, Yukon R exhibited significantly higher ΣPLFA and G^−^ compared to other genotypes. Yukon R also produced higher ΣB-PLFA, G^+^, fungal PLFA and eukaryotes however, statistically at par with DKC26-28RIB during 2016 (Table 4; Fig. 2). In 2017, Yukon R produced lower ΣPLFAs and ΣB-PLFA compared to 2016, but produced higher Σ PLFA, ΣB-PLFA, G^−^, G^+^, fungal PLFA and eukaryotes compared to other genotypes. DKC 23-17RIB produced minimum ΣPLFA, ΣB-PLFA, G^−^, G^+^, fungal PLFA and eukaryotes among all genotypes during 2017 (Table 4; Fig. 2). It is pertinent to mention here that higher F:B ratio was observed when Fusion RR was amended with low P_2_O_5_ manure compared to the lower F:B ratio recorded in the same genotype under control treatment (Table S4). Apparently, it seems that manure application influenced F:B ratio than genotypes.

### Association between soil biochemical properties and the active microbial population in podzolic soil

**Figure 3:**
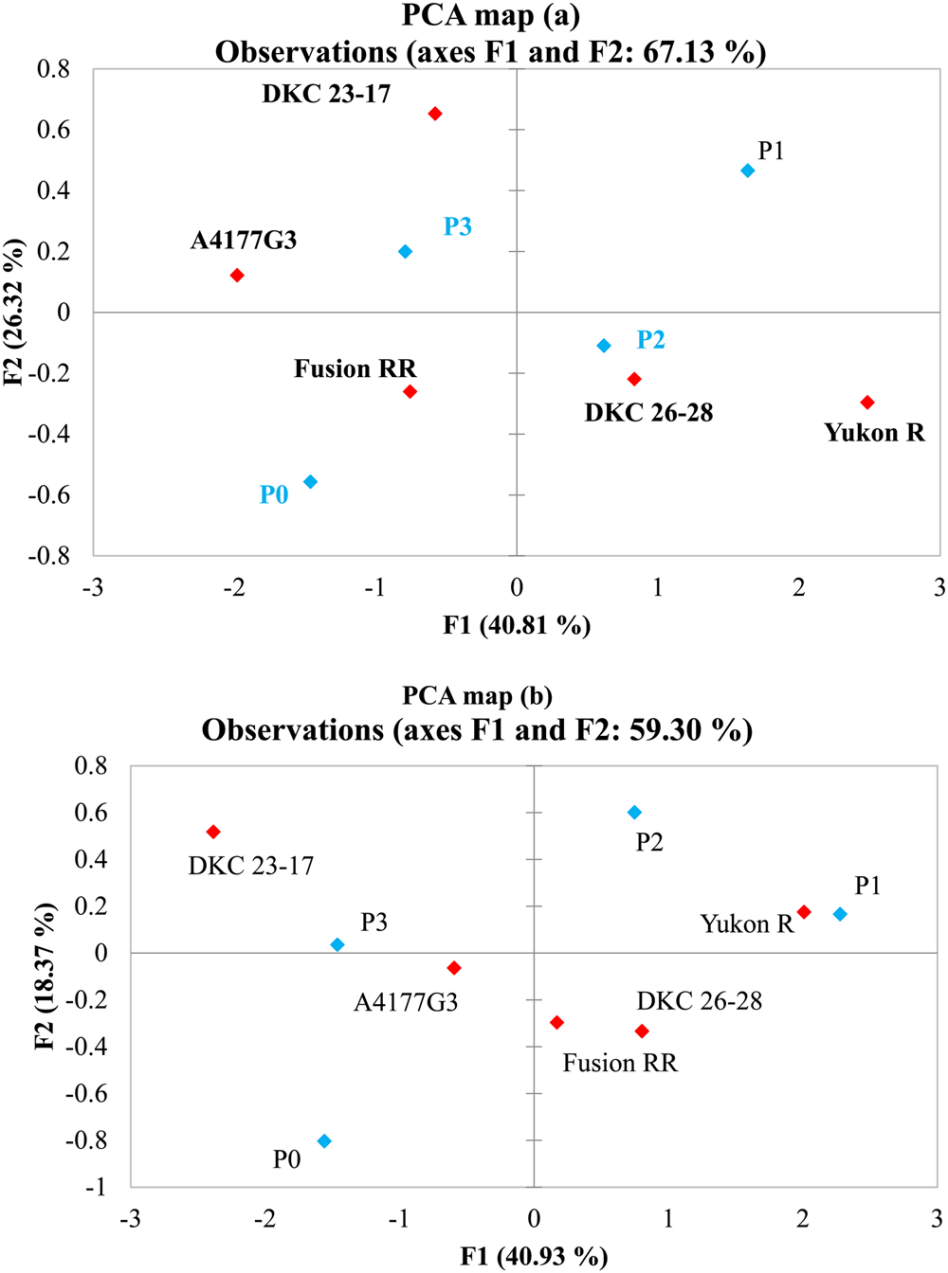
Observation plots of principal component analysis showing the segregation of five silage corn genotypes and four phosphorus amendments based on the centroids on the F1 and F2 axis during 2016 (**a**) and 2017 (**b**).

**Figure 4:**
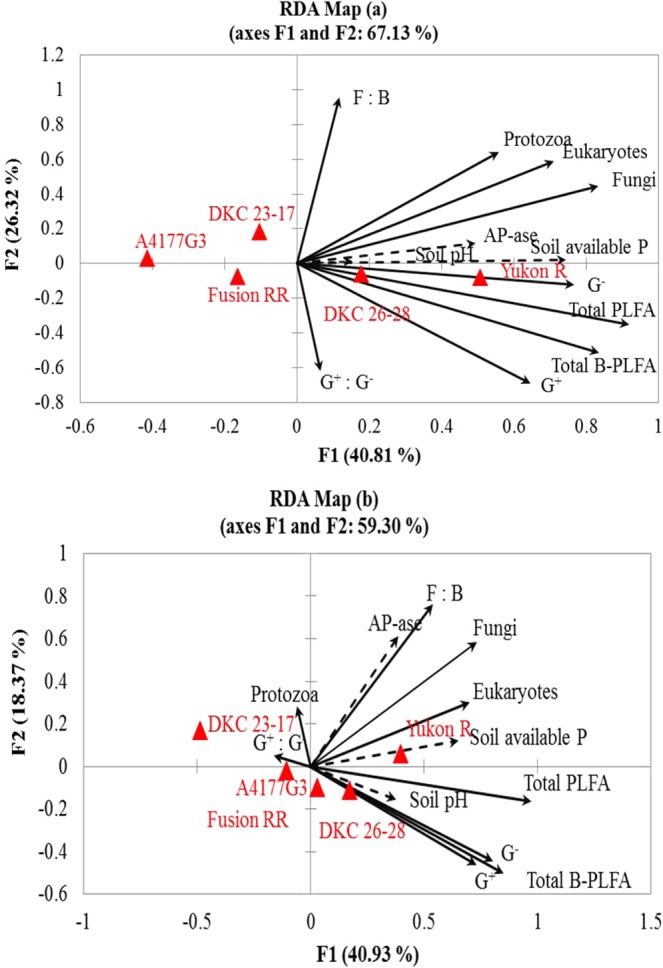
Redundancy analyses (RDA) of the correlations between biochemical attributes and microbial community composition in silage corn genotypes during 2016 (**a**), 2017 (**b**). Arrows with dotted line indicate the genotypes had strong and significant effects on biochemical attributes and arrows with solid line indicate strong and significant effects on microbial community composition (*P* < 0.05).

**Figure 5:**
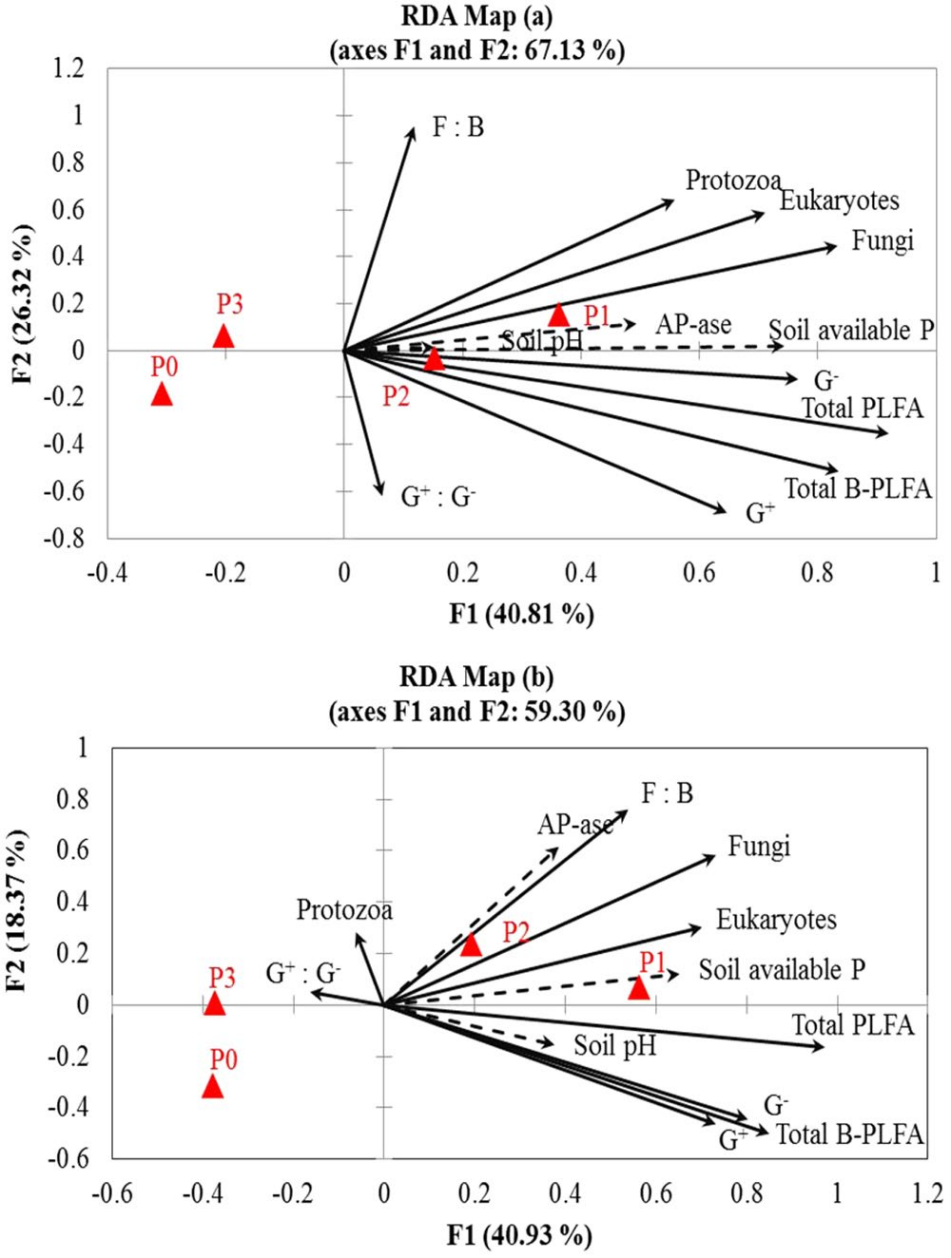
Redundancy analyses (RDA) of the correlations between biochemical attributes and microbial community composition between organic and inorganic phosphorus amendments  during 2016 (**a**), 2017 (**b**). Arrows with dotted line indicate organic and inorganic phosphorus sources had strong and significant effects on biochemical attributes and arrows with solid line indicate strong and significant effects on microbial community composition (*P* < 0.05).

Soil pH showed no significant relationship with AP-ase, SAP and soil microbial communities in 2016, whereas significant relationship of soil pH with SAP, G^+^, G^−^, ΣB-PLFA and ΣPLFA was observed in 2017 (Table 5). Positive and strong correlations between SAP, AP-ase, G^−^, eukaryotes, fungi, ΣB-PLFA and ΣPLFA were observed during 2016, whereas, significant and strong positive correlations between SAP, AP-ase, fungi, eukaryotes and F:B ratio were noted during 2017. SAP was significantly and positively correlated with G^−^, G^+^, eukaryotes, ΣB-PLFA and ΣPLFA. Likewise, AP-ase showed positive and strong correlation with G^−^, fungi, eukaryotes, ΣB-PLFA and ΣPLFA during 2016 and with fungi, eukaryotes and F:B ratio during 2017 (Table 5). PCA showed a clear separation of P amendments and genotypes in different quadrants in both years (Fig. 3a,b). Redundancy analysis was carried out between biochemical attributes, PLFAs, P amendments and silage corn genotypes for 2016 and 2017 (Fig. 4). Of the total variation between biochemical attributes and the active microbial community composition, 40.81% and 26.32% was observed in first and second axis respectively, during 2016 (Fig. 4a). In 2017, 40.93% and 18.37% of the total variation in the first and second axis was observed between biochemical, and PLFA parameters (Fig. 4b). Manure amendments (P_1_ & P_2_) showed strong and positive relationship with SAP, AP-ase, fungi, G^−^, total PLFA and total bacterial PLFA in both years which indicates that manure applications either with high or low P_2_O_5_ concentration significantly improved SAP, AP-ase and active microbial biomass, community and abundance (Fig. 5a,b). Similarly, Yukon R and DKC26-28RIB showed strong association with G^−^, total PLFA, total bacterial PLFA, fungi, eukaryotes, SAP, pH and AP-ase compared to other genotypes during 2016 (Fig. 4a). In 2017, Yukon R and DKC26-28RIB showed strong association with total PLFA, G^−^, total bacterial PLFA, soil pH, SAP, eukaryotes and fungi compared to other genotypes (Fig. 4b).

**Table 5:** Pearson’s correlation coefficients (r) showing the relationship between soil biochemical parameters and active microbial communities during 2016 and 2017.

Years		Ap-ase	SAP	pH
2016	Ap-ase		0.68***	−0.09^Ns^
	SAP			0.19^Ns^
	G +	0.17^Ns^	0.36**	0.12^Ns^
	G^−^	0.27*	0.57***	−0.00^Ns^
	F	0.35**	0.51***	0.21^Ns^
	P	0.11^Ns^	0.21^Ns^	0.04^Ns^
	E	0.31*	0.40**	0.04^Ns^
	ΣB-PLFAs	0.26*	0.55***	0.07^Ns^
	ΣPLFAs	0.30*	0.59***	0.09^Ns^
	G + :G^−^	−0.05^Ns^	−0.09^Ns^	0.13^Ns^
	F:B	0.19^Ns^	0.11^Ns^	0.14^Ns^
2017	Ap-ase		0.30*	0.10^Ns^
	SAP			0.28*
	G+	−0.01^Ns^	0.39**	0.27*
	G^−^	0.08^Ns^	0.39**	0.30*
	F	0.48***	0.48***	0.15^Ns^
	P	0.10^Ns^	−0.09^Ns^	0.03^Ns^
	E	0.44***	0.39**	0.08^Ns^
	ΣB-PLFAs	0.04^Ns^	0.43***	0.32*
	ΣPLFAs	0.25^Ns^	0.52***	0.30*
	G+:G^−^	−0.10^Ns^	−0.04^Ns^	−0.06^Ns^
	F:B	0.49***	0.38**	0.08^Ns^

Abbreviations: Ap-ase = acid phosphatase, SAP = soil available P, G^+^ = gram positive, G^−^ = gram negative, F = fungi, P = protozoa, E = eukaryotes, Σ B-PLFAs = total bacterial PLFAs, Σ PLFAs = total PLFAs, G^ +^ : G^−^ = gram positive/gram negative ratio, F: B = fungi/bacteria ratio. *** significant at *p* < 0.001, ** significant at *p* < 0.01, *significant at *p* < 0.05, NS = Non-significant.

## Discussion

### Biochemical attributes

Soil enzymes are instrumental in catalyzing organic matter decomposition reactions and nutrients turnover. Therefore, they can be used as early indicators of land use changes and soil health determinants caused by agricultural practices^40^. For example, AP-ase and other enzymes activities fluctuate rapidly due to fertilizers application, complex rhizosphere processes, biological properties, and environmental conditions^41^. Manure contains organic P that is mineralized by phosphatases to IP before plants uptake that; soil microbial and enzyme activities significantly affect P hydrolysis^23^. Soil and plants microorganisms have evolved several mechanistic means to solubilize bound inorganic P and mobilize organic P through exudation of secondary metabolites such as oxalate and citrate^42^. In the present study, it seems that root exudates potential vary among the silage corn genotypes, which affected the solubilization of carbon, nitrogen and phosphorus, and shaped the microbial community to decompose the manure by secretion of various enzymes. Therefore, we observed higher AP-ase in Yukon R rhizosphere amended with high P_2_O_5_ manure and lowest in DKC 23-17RIB genotype cultivated in the control treatment (Table 2). Previous studies also reported that AP-ase activity was stimulated by root exudates and manure application^43,44^. Increased AP-ase and SAP with high P_2_O_5_ manure application in the present study could be attributed to enhanced microbial activities by providing organic sources, such as C, N and P^1,16,33^. Results of the present study also demonstrate that high P_2_O_5_ manure amendment enhanced AP-ase and SAP availability compared to IP and control treatment (Table 2). Pearson’s correlation also showed significant and positive correlation between AP-ase activity and SAP (Table 5).

Soil pH determines the availability of macro and micronutrients^42^, modify soil physiochemical properties and microbial community composition^45^. In acidic soils or near neutral soils, manure generally maintain or increase soil pH through liming effect^46^. Whereas, Dong *et al*.^12^ observed that inorganic fertilizers application to soil (alkaline in nature) may return some alkaline substance which may lead to increased soil pH. In the present study, interactive effects of inorganic phosphorus (P_3_) and silage corn genotypes increased the soil rhizosphere pH, although it was statistically at par with high P_2_O_5_ manure (P_1_ treatment) (Table 1), suggesting that either silage corn roots or microbial communities released specific organic compounds in the rhizosphere that might have altered the soil pH. Shen *et al*.^47^ also observed that plant roots exude organic metabolites and some specific signaling compounds, which are key drivers of various rhizosphere processes. Additionally, preferential uptake of cations and anions by different crop genotypes or species under different nutrient regimes might have altered soil rhizosphere pH^42,48^.

### Soil microbial PLFA

**Figure 6:**
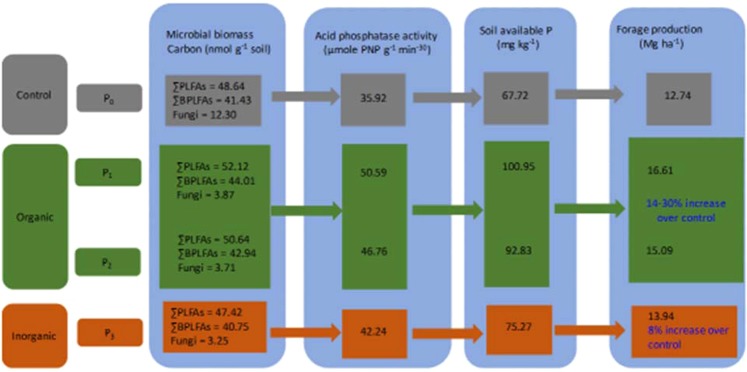
A stylized framework illustrating the effects of organic and inorganic phosphorus amendments stimulating total microbial biomass and acid phosphatase activities, which enhance soil available phosphorus and consequently silage corn forage production. The values are averaged over two growing seasons (2016 & 2017), whereas percent increase forage production in organic and inorganic P amendments was calculated keeping control as base value (P_1_ – P_0_/P_0_ × 100; P_2_ – P_0_/P_0_ × 100; P_3_ – P_0_/P_0_ × 100). The green and orange background shows increase in microbial biomass carbon, acid phosphatase activity, soil available P and forage production in organic and inorganic phosphorus amendments over control.

Several PLFA based studies reported significant differences in soil microbial community composition and abundance under different fertilizer management practices^49,50^. Soil microbial community (SMC) are sensitive to external application of N and P^51,52^. Phosphorus fertilization significantly increased microbial community, diversity and abundance in pastures tropical forest, and grasslands ecosystems^51,52^. We observed higher ΣPLFA, ΣB-PLFA, and fungi in high P_2_O_5_ manure (P_1_ treatment) than the control and IP treatment (Fig. 1), additionally, organic P amendments stimulated total microbial biomass and AP-ase activities which enhance SAP and silage corn forage production (Fig. 6). Agricultural soils are generally C limited and manure application stimulate the growth of microbes by enhancing SOC, N and P pool in soil (Tables 3, 4), which most probably serve as major energy sources for microorganisms^53^. In the present study, it seems that high P_2_O_5_ manure application not only provided readily available substrate for the microbial community rather other macro and micronutrients particularly high CN (Tables 3, 4), whereas, inorganic P fertilization probably contains small labile organic C which might be not enough to stimulate the substantial growth of microorganisms^31^. In typical intensively cultivated agricultural soils, bacterial biomass was higher while fungal biomass was lower^54^. It has been observed that manure application significantly increased G^−^ relative to G^+^ bacterial biomass, probably due to higher availability of C (Tables 3, 4) over longer periods during the growing season than inorganic fertilization^50^. Moreover, higher population of G^−^ bacteria usually occur when there is a shift from nutrient deficient condition to nutrient rich conditions, and this pattern was observed in soil amended with P fertilizers^55^. In the present study, we found significant differences in G^−^ bacterial biomass with low nutrient to rich nutrient manure and IP fertilizer amendment. High P_2_O_5_ manure amendment proliferated G^−^ bacterial biomass and was higher than control and IP application in both years (Fig. 1). Role of fungi in agricultural ecosystems is pivotal, particularly in C and nutrient cycling and are sensitive to fertilizers application^31^. In general, fungal to bacteria ratio is relatively low and is common phenomenon in agricultural ecosystems due to intensive physical disturbance and changes in amount, type and source of fertilizers inputs^54,56^. In the current experiment, we observed similar pattern of reduced F/B ratios from 0.087–0.094 during 2016 and 0.072–0.084 during 2017 with organic and inorganic P amendments in podzols (Table 6). Our results substantiate the findings of^57^ who reported lower values for inorganic fertilizers than manure amendments. Particularly, organic amendment (manure) substantially enhanced growth of fungi and thus increased F/B ratios whereas, inorganic mineral source reduced the F/B ratio^58^. Higher fungal PLFA in manure than in IP fertilizer amended plots (Fig. 1), might be due to additional C added by manure amendment which served as a major source of energy for fungi whereas, inorganic fertilization reduced fungal biomass due to small labile organic C^51^. In crux, P_1_ treatment (organic P amendment) exhibited higher total microbial biomass (52.12 nmol g^−1^ soil), AP-ase activity (50.59 µmol PNP g^−1^ min^−30^), SAP and consequently 30% higher forage production than control, whereas, inorganic P amendment showed 47.42 nmol g^−1^ soil total microbial biomass, 42.24 µmol PNP g^−1^ min^−30^ AP-ase activity, 75.27 mg kg^−1^ SAP and contributed 8% increase in forage production over control (Fig. 6). Enhanced soil microbial biomass, AP-ase, and SAP resulted in higher forage production in organic amended soils compared to control or inorganic P. Organic P amendments increased 14–30% forage production of silage corn whereas only 8% in case of inorganic P as (Fig. 6). Due to the complex soil rhizosphere system and presence of multiple microbial groups, relationship between soil microbes, their abundance and function is not straight forward^59^. Agricultural management practices may change the dynamics of soil microbial community composition, and function, and eventually crop growth and productivity^36,60,61^. For instance, different plant species and genotypes release significant number of secondary metabolites in the rhizosphere, which spur the growth of dormant microbial species^62,63^ A considerable portion (up to 21%) of photosynthetic C as root exudates in the form of soluble sugars, amino acids, or secondary metabolites are utilized by the SMC in the plants rhizosphere^64–66^. Furthermore, a variety of rhizosphere exudates released by plant roots attract specific microbes resulting in alteration of microbial communities and their abundance^67,68^, as observed in maize genotypes^69,70^. In the present study, Yukon R might have released some specific organic compounds to attract and select specific microbes, which in turn altered microbial composition and abundance, consequently exhibiting higher ΣPLFA, ΣB-PLFA, and fungi compared to the other genotypes (Table 7). Our results substantiate the findings of^71^, who reported substantial variation in bacterial richness, diversity and relative abundance in the maize rhizosphere of different inbred lines. Moreover, plants release specific secondary metabolites and organic compounds that shape and drive the microbial structure^34–36^ Furthermore, we observed strong association between ΣB-PLFA, ΣPLFA, soil enzyme (AP-ase), and SAP with Yukon R genotype during 2016. In 2017, Yukon and DKC 26-28RIB showed similar trend with microbial communities and soil biochemical attributes (Fig. 4a,b). To further elucidate and underpin the role of organic compounds released by silage corn genotypes in selecting and attracting specific microbial community and function should be the direction of future research when cultivated on podzolic soils under cool climatic conditions in boreal ecosystem.

**Table 6:** Amounts of total, bacterial, protozoa, eukaryotes fungal, gram-positive and gram-negative bacterial PLFAs (nmol g^−1^ soil) under organic and inorganic phosphorus amendments at black layer stage during 2016 and 2017.

	P sources	Σ PLFAs	Σ B-PLFAs	G-	G +	F	P	E	G^+^:G^−^	F:B
2016	P_0_	48.309 ^bc^	41.026 ^ab^	21.262 ^bc^	19.764 ^a^	3.390 ^b^	1.421 ^a^	2.471 ^b^	0.938 ^a^	0.082 ^a^
	P_1_	51.605 ^a^	43.307 ^a^	22.942 ^a^	20.365 ^a^	3.975 ^a^	1.509 ^a^	2.812 ^a^	0.883 ^a^	0.094 ^a^
	P_2_	50.81 ^ab^	42.819 ^a^	22.024 ^ab^	20.795 ^a^	3.725 ^ab^	1.545 ^a^	2.720 ^ab^	0.954 ^a^	0.087 ^a^
	P_3_	47.412 ^c^	39.793 ^b^	20.533 ^c^	19.259 ^a^	3.487 ^b^	1.515 ^a^	2.617 ^ab^	0.947 ^a^	0.087 ^a^
2017	P_0_	48.149 ^c^	42.253 ^bc^	21.555 ^b^	20.698 ^bc^	2.691 ^c^	1.809 ^a^	1.394 ^c^	0.960 ^a^	0.063 ^c^
	P_1_	52.647 ^a^	44.719 ^a^	23.101 ^a^	21.618 ^a^	3.778 ^a^	1.776 ^a^	2.373 ^a^	0.938 ^a^	0.084 ^a^
	P_2_	50.462 ^b^	43.079 ^b^	22.003 ^b^	21.076 ^ab^	3.700 ^a^	1.730 ^a^	1.952 ^b^	0.958 ^a^	0.086 ^a^
	P_3_	47.434 ^c^	41.517 ^c^	21.335 ^b^	20.182 ^c^	3.025 ^b^	1.763 ^a^	1.127 ^c^	0.948 ^a^	0.072 ^b^

Abbreviations: Σ B-PLFAs = total bacterial PLFAs, Σ PLFAs = total PLFAs, G^+^  = gram positive, G^−^ = gram negative, F = fungi,

P = protozoa, E = eukaryotes, G ^+^ : G^−^ = gram positive/gram negative ratio, F: B = fungi/bacteria ratio, NS = non-significant.

(Common letter means do not differ significantly at p < 5%).

**Table 7:** Variation in rhizosphere total, bacterial, protozoa, eukaryotes fungal, gram-positive and gram-negative bacterial PLFAs (nmol g^−1^ soil) of silage corn genotypes at black layer stage during 2016 and 2017.

	Genotypes	Σ PLFAs	ΣB-PLFAs	G^−^	G^+^	F	P	E	G^+^:G^−^	F:B
2016	Fusion RR	47.39 ^c^	39.77 ^b^	19.00 ^d^	20.77 _ab_	3.56 ^bc^	1.48NS	2.56 ^ab^	1.09 ^a^	0.09 NS
	Yukon R	54.87 ^a^	46.50 ^a^	24.76 ^a^	21.74 ^a^	4.02 ^a^	1.55	2.78 ^a^	0.88 ^b^	0.086
	A4177G3RIB	45.69 ^c^	38.63 ^b^	20.59 ^c^	18.04 ^c^	3.26 ^c^	1.39	2.39 ^b^	0.87 ^b^	0.087
	DKC 23-17RIB	47.93 ^c^	40.00 ^b^	21.19 ^c^	18.81 ^bc^	3.63 ^abc^	1.54	2.75 ^a^	0.89 ^b^	0.090
	DKC 26-28RIB	51.76 ^b^	43.75 ^a^	22.89 ^b^	20.85 ^ab^	3.74 ^ab^	1.50	2.77 ^a^	0.91 ^b^	0.085
2017	Fusion RR	49.89 ^bc^	43.09 ^ab^	22.05 ^ab^	21.04 ^a^	3.34 ^bc^	1.72NS	1.72 ^ab^	0.95NS	0.077 ^ab^
	Yukon R	51.69 ^a^	44.29 ^a^	22.85 ^a^	21.43 ^a^	3.69 ^a^	1.79	1.91 ^a^	0.93	0.083 ^a^
	A4177G3RIB	49.00 ^c^	42.58 ^b^	21.60 ^bc^	20.98 ^a^	3.13 ^cd^	1.81	1.48 ^b^	0.97	0.073 ^b^
	DKC 23-17RIB	46.71 ^d^	40.58 ^c^	20.84 ^c^	19.74 ^b^	2.86 ^d^	1.79	1.45 ^b^	0.95	0.070 ^b^
	DKC 26-28RIB	51.06 ^ab^	43.89 ^ab^	22.64 ^a^	21.25 ^a^	3.46 ^ab^	1.71	1.98 ^a^	0.94	0.079 ^ab^

Abbreviations: Σ B-PLFAs = total bacterial PLFAs, Σ PLFAs = total PLFAs, G^+^  = gram positive, G^−^ = gram negative, F = fungi,

P = protozoa, E = eukaryotes, G + : G- = gram positive/gram negative ratio, F: B = fungi/bacteria ratio, NS = non-significant.

(Common letter means do not differ significantly at p < 5%).

## Conclusion

In two years field research trial, organic and inorganic P amendments significantly influenced the soil biochemical attributes, soil microbial community composition and abundance in silage-corn production systems in podzolic soil under boreal climate. Results suggest that P1 treatment (DM with high P_2_O_5_, N and C) significantly enhanced active microbial community composition and abundance, AP-ase, and SAP compared to control and inorganic P amendment. Redundancy analyses also demonstrated strong association among P1 and P2 treatments, AP-ase, SAP, total bacterial PLFA, total PLFA and fungi suggesting that organic P amendment could be a sustainable management practice effective strategy for attaining higher forage yield of silage corn in podzolic soils under boreal climate. Results further suggest that Yukon R and DKC 26-28RIB exhibited higher total PLFA, total bacterial PLFA and fungal biomass, in their soil rhizospheres and showed superior agronomic performance compared to the other genotypes. Taking all together, we can conclude that DM amendment could be a sustainable nutrient management strategy to enhance soil quality, health and forage production of silage corn in podzol soils under boreal climate. We also argue that Yukon R and DKC 26-28RIB genotypes could be good fit to attain higher forage production in boreal climate.

## Material and Methods

### Experimental design

**Figure 7:**
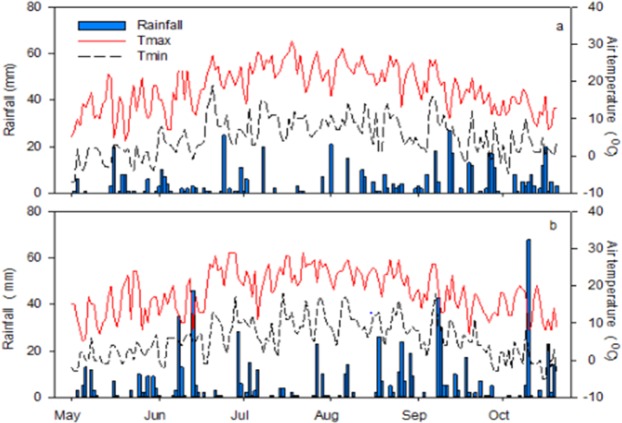
Weather conditions at Pynn’s Brook Research Station, Pasadena during 2017 (**a**) and 2016 (**b**) growing season.

**Figure 8:**
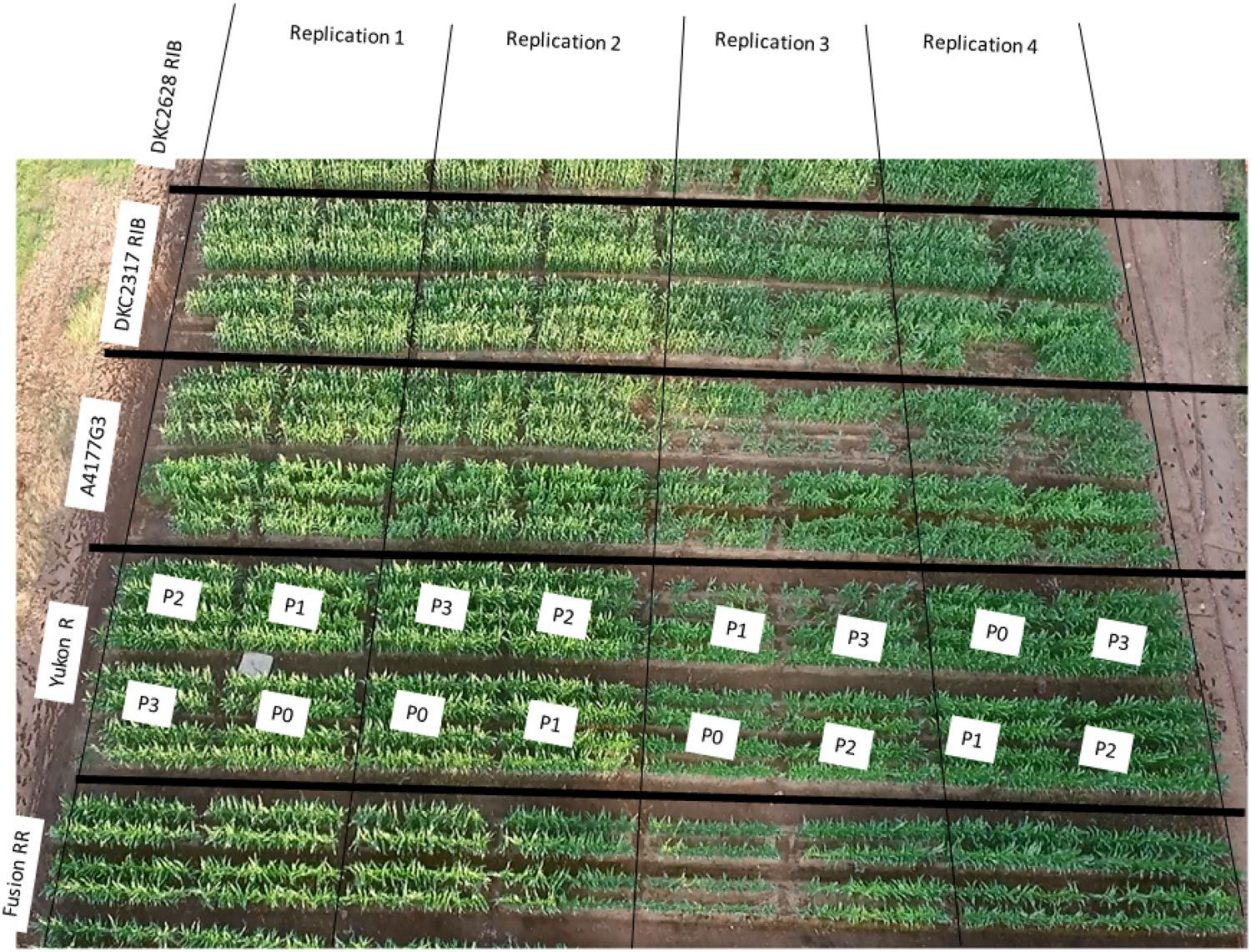
Aerial view of experimental field layout showing the five silage corn genotypes, four phosphorus treatments and randomization of phosphorus treatments as an example in Yukon-R.

Field research trials were conducted at Pynn’s Brook Research Station (49.50° N, 57.33° W), Newfoundland and Labrador (NL), Canada during 2016 and 2017 growing season. Soil texture was determined to be characterized with 82% sand, 12% silt, and 6% clay particles (loamy-sand). Soil analyses prior to crop seeding during both study years are given in Table 8. Mean average temperature and rainfall during 2016 and 2017 growing seasons were 12.2 °C, 11.8 °C, and 704 mm and 496 mm, respectively (Fig. 7). The experimental treatments were: 1) DM with high P conc. (P_1_); 2) DM with low P conc. (P_2_); 3) Inorganic P (P_3_); 4) control (P_0_) and five silage-corn cultivars (Fusion RR, Yukon R, A4177G3RIB, DKC 23-17RIB, and DKC 26-28RIB). Experimental plots were fertilized with dairy manure @ 30,000 L ha^−1^ considering the local dairy farmer’s practice and mixed in the upper 15-20 cm soil one day before seeding. Triple super phosphate (TSP; 0-46-0) was used as IP source @ 110 kg ha^−1^. Ammonium nitrate (AN) and murate of potash (MOP) were used as nitrogen (N), and potash (K) source to fulfill the mineral requirement of crop. Full K and half N was applied at seeding, while remaining half N was applied at twelve leaf stage following regional recommendation, soil test report and results obtained from manure analyses reports. It is pertinent to mention here that experimental treatment plots were geo-referenced to keep the same plots during both years of study to avoid any treatment effects. The individual experimental treatment plot was comprised of 3 × 5 m dimensions with four rows of silage corn, and plots were orientated in east-west directions. Experiments were laid-out in randomized complete block design (RCBD) with four replications; however, one replication was omitted from data analysis (Fig. 8). Manure with high and low P_2_O_5_ concentrations were chosen after analyzing 13 dairy farms manure samples as organic P source and designated as P_1_ (high P_2_O_5_) and P_2_ (low P_2_O_5_). Detailed dairy manure analysis reports are given in Table 9.

**Table 8:** Pre-seeding soil analyses reports of experimental site in 2016 and 2017.

Soil properties	2016	2017
Soil pH	6.4	6.8
Phosphorus (mg/L)	81	74
Potassium (mg/L)	38	49
Calcium (mg/L)	1256	1120
Magnesium (mg/L)	265	218
Organic matter (%)	2.98	3.01
Sulphur (mg/L)	14	15
Zinc (mg/L)	0.6	1.3
Copper (mg/L)	1.1	2.1
Sodium (mg/L)	7	6
Iron (mg/L)	150	130
Boron (mg/L)	0.1	0.2
Manganese (mg/L)	18	16
Aluminum (mg/L)	1507	1409

**Table 9:** Detailed analysis of manure with high and low phosphorus concentration used in this study during both study years.

	Manure with high P_2_O_5_		Manure with low P_2_O_5_	
Manure properties	2016	2017	2016	2017
Dry matter (%)	9.33	10.9	3.57	1.7
pH	6.8	6.8	7	7.1
Nitrogen (kg/1000 L)	1.5	1.9	0.6	0.5
Phosphate (P_2_O_5_ Kg/1000 L)	0.7	1.7	0.3	0.3
Total potassium (%)	0.33	0.37	0.12	0.12
Total calcium (%)	0.19	0.19	0.069	0.042
Total magnesium (%)	0.078	0.077	0.024	0.018
Total iron (ppm)	65	68	21	7
Total manganese (ppm)	26	21	9	5
Total copper (ppm)	5	4.5	23	20
Total zinc (ppm)	16	21	8	5
Total boron (ppm)	4	3.4	1.5	0.5
Total sodium (ppm)	788	904	271	241

### Soil sampling and analysis

A composite soil sample was prepared by gently removing the root adhered soil from three uprooted silage-corn plants in each treatment. The collected soil samples were placed in a cooler containing ice then transported immediately to boreal ecosystems research facility located at Grenfell Campus Memorial University. Composite soil samples were then sieved (2 mm mesh) to remove any debris or inert material and divided into three subsamples. First subsample was dried at 25 °C to determine soil pH and SAP. The second subsample was stored at 4 °C prior to measure the AP-ase activity in rhizosphere and third subsample was stored at −20 °C to determine the active microbial community composition and abundance using phospholipid fatty acid analyses (PLFAs).

### Biochemical attributes

Soil pH was determined from air dried soil with calcium chloride (0.01 M) in 1:2 ratio. Soil solution was then mixed for 30 min on an orbital shaker (Innova™ 2300 Platform Shaker, NB, USA) at 120 rpm, then allowed to stand for 1 h and pH readings were recorded with Mettler Toledo, Canada pH meter^72^. AP-ase activity was measured through determination the p-nitrophenol-phosphate (PNP) as described by^73^. One mL of 0.09 M citrate buffer was used to extract 100 mg soil sample in 15 mL polypropylene tubes followed by centrifugation at 5000 rpm (10 min). An aliquot (50 µL) of the supernatant was added to 96-well microplate along with PNP (50 µL) and citrate buffer (30 µL). After incubation at 37 °C for 30 min, NaOH (20 µL) was dispensed in all wells to terminate the further reaction followed by absorbance recording (450 nm) with BioTek Cytation 3 spectrophotometer. The absorbance was used to calculate the AP-ase in the sample and the values expressed in µM p-nitrophenol g^−1^ soil min^−30^. SAP was analyzed using the extraction method described by^74^. Soil was extracted with Mehlich-3 solution in 1:10 proportion in 50 mL Erlenmeyer flasks. The resulting mixture was then agitated on an orbital shaker at 120 rpm (Innova™ 2300 Platform Shaker, NB, USA) for 5 min. The solution was filtered using Whatman 42 filter papers (Sigma Aldrich, ON. Canada). Aliquots of the filtrate was diluted 50 times then analyzed with an AA3 Continuous Flow Analytical System (AA3HR, SEAL Analytical USA) to determine SAP (mg/kg) level in the root rhizosphere.

### Active microbial community assessment

The phospholipid fatty acid (PLFAs) concentrations (nmol g^−1^) were recorded to assess the active microbial community and abundance in soil samples^75,76^. Total microbial mass was estimated using the total concentrations of PLFAs (nmol g^−1^). The PLFAs were then categorized into different taxonomic groups following the published literature on biomarkers (Table 10)^77^. For this purpose, the 4 g soil was extracted with chloroform methanol (10 mL; 2:1 v/v) in 20 ml glass vials. The samples were homogenized with a sonicator for 5 minutes (amplitude 50; pulse on time: 5 seconds; and pulse off time; 10 seconds) while being cooled simultaneously in an ice bath. The sonicated samples were allowed to settle down at room temperature (24 h). The collected supernatants were then filtered and dried under a gentle stream of nitrogen. Extracted lipids were dissolved in chloroform (2 mL) and neutral lipids, glycolipids and phospholipids were fractioned by solid phase on silicic acid columns (Discovery® DSC- Si SPE tube, 50 µm, 70 Å, 100 mg/mL; ThermoScientific, Brampton, Canada) extraction using chloroform (2.5 mL), acetone (4 mL) and methanol (2.5 mL). The separated phospholipids were then dried under a nitrogen gas stream. The phospholipids were dissolved in methyl tertiary-butyl ether (500 µL) and 100 µL aliquots placed in a screw-cap vials along with trimethyl sulfonium hydroxide (50 µL) as derivatization agent to convert the fatty acids to FAMEs. The sample was then vortexed, mixed (30 sec) for reaction (30 min). Methyl nonadeconate (10 µL; 19:0 @ 160 µg mL^−1^) was used as internal standard and the samples analyzed with GC-FID (gas chromatography-flame ionization detection) or GC coupled to a tripple quadrouple tandem mass spectrometer (GC-GC/MS-MS) by Trace 1300 gas chromatograph (TSQ 8000 mass spectrometer; ThermoScientific, Brampton, Canada).

**Table 10:** Phospho lipid fatty acid (PLFA) used as microbial biomarkers.

Taxonomic group	Biomarkers	References
Gram positive (G^+^)	C14_0	^78^
	i-C15_0	^79,80^
	a-C15_0	^79,80^
	C15_0	^81,82^
	i-C16_0	^79,80^
	C16_0	^83,84^
	C16_1n-7	^79,85^
	i-C17_0	^79,80^
	C17_0	^81,82^
	C18_0	^84,85^
	C18_1n-9cis	^80,85^
Gram Negative (G^−^)	2OH_C10_0	^86^
	2OH_C12_0	^86^
	C16_0	^83,84^
	C16_1n-7	^84,85^
	3OH_C12_0	^87^
	cycloC17_0	^79,80^
	C18_0	^84,85^
	C18_1n-9_trans	^88^
	C18_1n-9cis	^80,85^
	3OH_C14_0	^82^
	cycloC19_0	^79^
	C14_1n_5	^80^
	C17_1n_7	^76^
Fungi	C18_1n_9cis	^80,85^
	C18_2n_6cis	^80,89^
	C18_3n_3	^84,90^
	C20_1n_9	^16,90^
Protozoa	C20_0	^91^
	C20_3n_6	^92^
	C20_4n_6	^93^
Eukaryotes	C18_2n_6cis	^80,89^
	C21_0	^94^

### Phospholipid fatty acid (PLFA) analysis with GC-FID

The methylated fatty acids were fractioned with a DB-23 high resolution column (Agilent Technologies, Canada) by supplying helium as carrier (continuous flow rate of 1 mL min^−1^). Tri-plus auto sampler was used to inject 1 μL of each samples by splitless GC injector mode. The oven temperature varied in time spans (50 °C min; rise to 175 °C considering 20 °C min^−1^; held for 1 min followed by another rise to 230 °C at the rate of 4 °C and held for 5 min). The commercial standards (NIST database; Thermo Scientific, ON. Canada; Supelco-37 component fatty acid methyl ester mix; bacterial acid methyl ester mixed Sigma Aldrich, ON Canada) were used to identify the methylated PLFAs considering retention time and mass spectra. Methylated PLFA were quantified using internal standards and the results expressed in nmol g^−1^ soil. We have identified 47 PLFAs whereas, 27 were used to identify and quantify active microbial composition and abundance (Table 10)

### Statistical analysis

The effects of P amendments and silage corn genotypes on biochemical attributes and active soil microbial community composition and abundance were assessed. First, the Shapiro-Wilk test was performed to check the normality of the data set on each variable under study. The statistics calculation was conducted using Statistix-10 (Analytical Software, US) with the level of significance set at P < 0.05. P-value smaller than 0.05 indicates that the possibility of assumption is greater than 95%. Association between soil biochemical parameters and microbial community structure was quantified by Pearson’s correlation analyses. Principal component analyses (PCA) and Redundancy analyses (RDA) was performed using XLSTAT (Addinsoft, New York, USA), to assess the effects and relationships of silage corn genotypes and P amendments on soil biochemical parameters and the active soil microbial populations. Sigma plot 14.0 (Systat Software Inc.) and XLSTAT (Addinsoft, New York, USA) were used to make graphs.

## Supplementary information

Supplementary table
