# Supplementary material for: The effects of organic and inorganic phosphorus amendments on the biochemical attributes and active microbial population of agriculture podzols following silage corn cultivation in boreal climate
Source: Sci Rep. 2019 Nov 21;9:17297. doi: 10.1038/s41598-019-53906-8 (PMC6872752; doi:10.1038/s41598-019-53906-8)
Supplement: Supplementary file 1 — Supplementary table [file 41598_2019_53906_MOESM1_ESM.docx]

**The effects of organic and inorganic phosphorus amendments on the biochemical attributes and active microbial population of agriculture podzols following silage corn cultivation in boreal climate**

Waqas Ali^a^, Muhammad Nadeem^a^, Waqar Ashiq^a^, Muhammad Zaeem^a^, Syed Shah Mohioudin Gilani^a^, Sanaz Rajabi Khamseh^b^, Thu Huong Pham^a^, Vanessa Kavanagh^c^, Raymond Thomas^a^, Mumtaz Cheema^a*^

^a^School of Science and the Environment, Grenfell Campus Memorial University of Newfoundland, Corner Brook, Canada. A2H 5G4

^b^Shahrekord University, Rahbr Blvd, Shahrekord Chaharmahal and Bakhtiari, Iran.

^c^Department of Fisheries and Land Resources, Government of Newfoundland and Labrador, Pasadena, Canada.

*Corresponding author: Dr. Mumtaz Cheema, Email: mcheema@grenfell.mun.ca

**Table S1:** Analysis of variance for genotypes, phosphorus amendments and their interaction on biochemical attributes.

|  | Biochemical Parameters | Genotypes (G) | Phosphorus amendments (P) | G x P | Coefficient of variation% |
| --- | --- | --- | --- | --- | --- |
| 2016 | Soil pH | *** | * | * | 0.9 |
|  | Acid phosphatase activity | *** | *** | *** | 2.72 |
|  | Soil available P | *** | *** | ** | 8.07 |
|  | Total soil nitrogen | * | *** | NS | 13.90 |
|  | Total soil carbon | NS | *** | NS | 9.79 |
| 2017 | Soil pH | *** | ** | ** | 2.78 |
|  | Acid phosphatase activity | NS | *** | NS | 21.73 |
|  | Soil available P | *** | *** | NS | 21.37 |
|  | Total soil Nitrogen | * | *** | NS | 14.85 |
|  | Total soil carbon | NS | *** | NS | 12.51 |

*** Significant at *p < 0.001*, ** significant at *p < 0.01*, *significant at p *< 0.05*, NS= Non-significant.

**Table S2:** Acid phosphatase activities (AP-ase; µmole PNP g^-1^ min^-30^) influenced by silage-corn genotypes (A) and organic and inorganic phosphorus amendments on soil available phosphorus (mg kg^-1^) during 2017 (B).

|  | Fusion RR | Yukon R | A4177G3 RIB | DKC 23-17RIB | DKC26-28RIB |  |
| --- | --- | --- | --- | --- | --- | --- |
| A |  |  | Soil acid phosphatase activity | |  | Means |
| P_0_ | 40.80 | 62.96 | 62.18 | 68.58 | 55.88 | 58.08 ^C^ |
| P_1_ | 78.71 | 95.07 | 86.29 | 76.75 | 80.47 | 83.45 ^A^ |
| P_2_ | 67.30 | 94.49 | 81.04 | 75.09 | 70.30 | 77.64 ^AB^ |
| P_3_ | 67.75 | 69.54 | 74.71 | 69.36 | 62.97 | 68.86 ^BC^ |
| Means | 63.64 ^B^ | 80.51 ^A^ | 76.05 ^AB^ | 72.44 ^AB^ | 67.40 ^B^ |  |
| B Soil available phosphorus | | | | | | |
| P_0_ | 73.12 | 119.59 | 66.62 | 49.86 | 67.34 | 75.3 ^B^ |
| P_1_ | 112.92 | 139.75 | 88.17 | 67.05 | 116.9 | 104.96 ^A^ |
| P_2_ | 100.34 | 132.7 | 78.22 | 74.72 | 114.26 | 100.05 ^A^ |
| P_3_ | 81.99 | 121.1 | 72.61 | 55.09 | 57.08 | 77.57 ^B^ |
| Means | 92.09 ^B^ | 128.29 ^A^ | 76.41 ^BC^ | 61.68 ^C^ | 88.89 ^B^ |  |

Different superscripts indicate significant differences among treatment means at *p* < 0.05. P_0_ control, P_1_ manure with high P_2_O_5_, P_2_ manure with low P_2_O_5_, P_3_ inorganic P. Fusion RR, Yukon R, A4177G3RIB, DKC 23-17RIB, DKC26-28RIB are silage corn genotypes.

**Table S3:** Analysis of variance for genotypes, phosphorus amendments and their interaction on soil microbial population.

|  | Soil microbial | Genotypes | Phosphorus sources | G x P | Coefficient of |
| --- | --- | --- | --- | --- | --- |
|  | Community | (G) | (P) |  | Variation (%) |
| 2016 | G^+^ | * | NS | NS | 13.65 |
|  | G^-^ | *** | *** | NS | 6.97 |
|  | Fungi | * | * | NS | 14.02 |
|  | Protozoa | NS | NS | NS | 13.21 |
|  | Eukaryotes | * | NS | NS | 13.27 |
|  | Total bacterial PLFA | *** | * | NS | 8.47 |
|  | Total PLFA | *** | ** | NS | 7.47 |
|  | G^+^:G^-^ | *** | NS | NS | 13.97 |
|  | F:B | NS | NS | NS | 19.64 |
| 2017 | G^+^ | ** | ** | NS | 5.22 |
|  | G^-^ | *** | *** | NS | 4.67 |
|  | Fungi | *** | *** | ** | 12.2 |
|  | Protozoa | NS | NS | NS | 9.19 |
|  | Eukaryotes | NS | *** | NS | 30.47 |
|  | Total Bacterial PLFA | *** | *** | NS | 3.94 |
|  | Total PLFA | *** | *** | NS | 3.77 |
|  | G^+^:G^-^ | NS | NS | NS | 5.79 |
|  | F:B | * | *** | ** | 13.2 |

*** Significant at p < 0.001, ** significant at p < 0.01, *significant at p < 0.05, NS= Non-significant.

**Table S4:** Interactive effects of organic and inorganic phosphorus amendments and silage-corn genotypes on soil fungal PLFA (nmol g^-1^ soil) and F:B ratio during 2017.

|  | Fusion RR | Yukon R | A4177G3 RIB | DKC 23-17RIB | DKC26-28RIB |  |
| --- | --- | --- | --- | --- | --- | --- |
|  |  | Soil fungal PLFA | | |  | Means |
| P_0_ | 2.00 ^e^ | 3.42 ^bc^ | 2.07 ^e^ | 2.61 ^de^ | 3.34 ^c^ | 2.69 ^C^ |
| P_1_ | 4.07 ^ab^ | 4.08 ^a^ | 3.59 ^abc^ | 3.46 ^abc^ | 3.67 ^abc^ | 3.77 ^A^ |
| P_2_ | 4.06 ^ab^ | 4.06 ^ab^ | 3.59 ^abc^ | 3.21 ^cd^ | 3.56 ^abc^ | 3.70 ^A^ |
| P_3_ | 3.23 ^cd^ | 3.17 ^cd^ | 3.25 ^cd^ | 2.18 ^e^ | 3.27 ^cd^ | 3.02 ^B^ |
| Means | 3.34 ^BC^ | 3.69 ^A^ | 3.13 ^CD^ | 2.86 ^D^ | 3.46 ^AB^ |  |
| F:B ratio | | | | | | |
| P0 | 0.047 ^e^ | 0.078 ^abc^ | 0.049 ^de^ | 0.065 ^cd^ | 0.078 ^abc^ | 0.063 ^C^ |
| P1 | 0.091 ^ab^ | 0.089 ^ab^ | 0.081 ^abc^ | 0.081 ^abc^ | 0.080 ^abc^ | 0.084 ^A^ |
| P2 | 0.094 ^a^ | 0.091 ^ab^ | 0.084 ^ab^ | 0.078 ^abc^ | 0.080 ^abc^ | 0.086 ^A^ |
| P3 | 0.077 ^bc^ | 0.075 ^bc^ | 0.078 ^abc^ | 0.057 ^de^ | 0.076 ^bc^ | 0.072 ^B^ |
| Means | 0.077 ^AB^ | 0.083 ^A^ | 0.073 ^B^ | 0.070 ^B^ | 0.079 ^AB^ |  |

Different superscripts indicate significant differences among treatment means at *p* < 0.05. P_0_ control, P_1_ manure with high P conc., P_2_ manure with low P conc., P_3_ inorganic P. Fusion RR, Yukon R, A4177G3RIB, DKC 23-17RIB, DKC26-28RIB are silage corn genotypes.
